# Humor Production Promotes Creativity: The Mediating Role of Self-Efficacy and the Moderating Role of Fear of Authority

**DOI:** 10.3390/bs15081003

**Published:** 2025-07-23

**Authors:** Zhiwen Dong, Boxuan Han, Tianchen Yang, Shiqi Chen, Yi Cao, Yubo Hou

**Affiliations:** 1 School of Educational Science, Shanxi University, Taiyuan 030006, China; dongzhiwen0920@sxu.edu.cn; 2 School of Psychological and Cognitive Sciences, Peking University, Beijing 100871, China

**Keywords:** humor production, creativity, self-efficacy, fear of authority

## Abstract

We conducted four empirical studies to investigate how, why, and when humor production impacts people’s creativity. Study 1 (*N* = 175), using the cartoon humor production paradigm, found that humor production was positively associated with creativity. Study 2 (*N* = 243), using a new sample, found that self-efficacy mediated the relationship between humor production and creativity. Study 3 (*N* = 225), via a manipulation-of-mediation-as-a-moderator (MMM) design, manipulating participants’ self-efficacy, replicated the results of Study 2. Furthermore, Study 4 (*N* = 433), using a cross-lagged design and three-wave data, extended the theoretical model to the workplace, and further demonstrated that the fear of authority alleviated the indirect effect of humor production on people’s creativity. These findings are of theoretical and practical significance for our understanding of humor production and creativity.

## 1. Introduction

Humor is widely utilized in human social interactions. A sense of humor, a core personal characteristic (Martin & Ford, 2018), is defined as the habitual individual differences in all sorts of behaviors, experiences, affects, attitudes, and abilities relating to amusement, laughter, jocularity, and so on (Cao et al., 2023; Martin, 2001). Numerous studies have found that a sense of humor is positively associated with physical and mental health, interpersonal liking, and subjective well-being (Cao et al., 2025; Cao et al., 2024; Martin et al., 2003; Yue & Hiranandani, 2014). Recently, scholars have attempted to investigate the relationship between humor and creativity (Akben & Coskun, 2024). For example, previous research has revealed that people would feel less anxiety and produce more creative thinking when in humorous situations (Smith et al., 1971). Moreover, it has been evidenced that the adoption of humorous teaching methods could enhance students’ creativity within the classroom (Powell & Andresen, 1985). Most studies focused on humor appreciation and creativity, while few studies investigated the potential effect of the active expression of humor (i.e., humor production) on human creativity. Humor production could help individuals break existing social norms and constraints, which might serve as a significant predictor of creativity (Cao et al., 2023). In this line, this study aimed to explore whether, why, and when humor production influences human creativity.

### 1.1. Humor Production and Creativity

Sense of humor can be delineated into two distinct facets: humor appreciation and humor production, as proposed by Köhler and Ruch (Köhler & Ruch, 1996). Humor appreciation refers to the ability to discern and appreciate humor conveyed by others, while humor production involves actively expressing humor, encompassing actions such as retelling jokes to friends or creating original humorous content, such as funny videos posted online (Cao et al., 2023; Ruch & Heintz, 2019). Previous research has indicated that humor production was associated with positive personality traits, including traits like openness to experience, extroversion, and intelligence (Silvia et al., 2021), all of which have been identified as significant predictors of creativity (Ruch & Heintz, 2019). In this study, we aim to focus on humor production, an active expression of humor, so as to better understand the antecedents of human creativity.

Previous research has demonstrated that actively engaging in humor production can help people view problems from a novel and unconventional perspective (O’Quin & Derks, 1997). In the process of expressing humor, skills such as unconventional thinking and adopting new perspectives may serve as prerequisites for individuals to harness their creative abilities (Cade, 2003). Moreover, other research has extended this understanding by revealing a positive correlation between humor expression and creativity. Specifically, individuals who participated in improvisational comedy training exhibited more outstanding performances in creativity tasks than those who did not receive such training (Kudrowitz, 2010). In this line, humor production could act as both a direct contributor and a dynamically intertwined element of creative processes (Galloway, 1994; O’Quin & Derks, 1997). Based on the above, we put forward the following hypothesis:

**Hypothesis** **1.** *Humor production predicts creativity positively*.

### 1.2. The Mediating Role of Self-Efficacy

Self-efficacy refers to an individual’s belief in his or her ability to exert control over events that affect his or her life (Bandura, 1977). Schwarzer et al. further defined self-efficacy as an individual’s overall self-confidence when facing new environments or circumstances (Schwarzer et al., 1997). Previous research has found that self-efficacy plays a pivotal role in bolstering an individual’s persistence, task initiation, and creative thinking (Bandura & Locke, 2003). Thus, we propose that self-efficacy could explain why humor production increases people’s creativity.

On the one hand, humor production could enhance people’s self-efficacy. It has been evidenced that positive emotions are a crucial antecedent of self-efficacy (Fredrickson, 1998; Ouweneel et al., 2013). Previous research has found that the impact of a sense of humor on positive emotions primarily stems from humor production (Fu et al., 2024; Robert & Wilbanks, 2012). Specifically, humor production not only assists individuals in alleviating psychological stress and anxiety (Martin & Dobbin, 1989; Shi et al., 2025) and gaining social approval (Cann & Matson, 2014), but also leads to feelings of superiority and self-esteem (Cantor & Zillmann, 1973). According to social cognitive theory, these positive emotions in individuals contribute to positive self-evaluation, thereby enhancing self-efficacy, such as those who believe they outperform others having higher self-efficacy compared to those who perceive themselves as performing less effectively than others (Bandura, 1997). Consequently, individuals skilled in humor production are more likely to exhibit heightened positive emotions, thus fostering their self-efficacy. Moreover, previous research has found that a sense of humor was positively associated with self-efficacy (L. Hu et al., 2023).

On the other hand, self-efficacy plays a vital role in promoting people’s creativity (Bandura & Locke, 2003). People with high self-efficacy tend to perceive their tasks as more engaging, which motivates proactive exploration of novel ideas and enhanced creative expression. And they are more likely to adopt constructive coping strategies throughout the task completion process (Alipan et al., 2021; McBride & Ireland, 2016; Yang et al., 2010), which facilitates proactive problem-solving or help-seeking behaviors under challenging conditions. In contrast, individuals with low self-efficacy are prone to adopting negative coping strategies (e.g., indulging in fantasy, venting, self-blame, or seeking escape), thus leading to less creativity (Geng & Zhao, 2022; Lu, 2014). Furthermore, Robert and Wilbanks (Robert & Wilbanks, 2012) posited that humor production acts as an essential catalyst for fostering positive emotions, which, in turn, broadens individuals’ cognitive and behavioral capacities. These expanded capacities encourage individuals to transcend conventional thought and action patterns, thereby embarking on new, creative, and often unconventional avenues of thinking and behavior (Fredrickson, 1998). Based on the above, we propose the following hypothesis:

**Hypothesis** **2.** *Self-efficacy mediates the relationship between humor production and creativity*.

### 1.3. The Moderating Role of Fear of Authority

Humor production serves as a critical component of interpersonal communication, pervasively manifested in peer interactions, colleague collaborations, and cross-hierarchical organizational exchanges. Empirical evidence confirmed that humor is flourishing in a relatively equitable social hierarchy (Liao & Chang, 2006). However, when individuals fear authority, will their humor still be the same when facing leaders? The fear of authority refers to individuals’ fear of authoritative over others and their evaluation (Zi & Zhou, 2006). Influenced by collectivism and the culture of high power distance, authoritarian leadership, characterized by swearing absolute authority and demanding absolute obedience from subordinates, is a common leadership style in non-Western cultures (especially in China) (Ahmad Bodla et al., 2019). Specifically, individuals in the workplace will be significantly influenced by this leadership, which, in turn, may impinge upon their willingness and ability to express humor at work. Thus, humor production in the workplace may face heightened scrutiny (Ruch & Heintz, 2019).

Individuals with a high fear of authority are often reluctant when it comes to using humor as a means of communicating with their superiors or proposing innovative work concepts (Jenkins, 2014). In this line, it is difficult for them to produce humor to enhance their self-efficacy and creativity. In contrast, individuals with a low fear of authority are more likely to engage in more active and relaxed social exchanges with their superiors in the workplace and are more willing to share their novel ideas (Bitterly & Schweitzer, 2019). Therefore, they are more likely to experience a heightened sense of self-efficacy when producing humor, further fueling their creative endeavors (Bandura, 1977). Thus, we propose a moderated mediation model wherein humor production potentiates creativity through self-efficacy, with the indirect effect being attenuated under conditions of elevated hierarchical anxiety (i.e., fear of authority). Based on the above, we put forward the following hypothesis:

**Hypothesis** **3.** *Fear of authority moderates the mediating effect of self-efficacy between humor production and creativity. Specifically, the indirect effect in the low fear of authority is stronger than that in the high fear of authority*.

### 1.4. The Present Research

We conducted four empirical studies to test our conceptual framework regarding the process through which humor production acts as a promoting factor in increasing individuals’ creativity. Specifically, Study 1 first established an initial relationship between humor production and creativity using two-wave data. Study 2 further explored the mediating role of self-efficacy through a new sample and a cross-sectional design. Based on that, Study 3 aimed to replicate the findings of Study 2 through manipulating self-efficacy. Moreover, Study 4 aimed to extend the theoretical model to the workplace, and validate the moderating role of fear of authority in the mediating role of self-efficacy between humor production and creativity.

## 2. Study 1

Study 1 intended to provide preliminary evidence for Hypothesis 1 (i.e., humor production could positively predict creativity).

### 2.1. Methods

#### 2.1.1. Participants and Procedure

We recruited 220 college students through the anonymous Bulletin Board System (BBS) of Peking University and WeChat Moments. A total of 45 participants failed the quality check question and thus were excluded, resulting in a final sample of 175 participants (54 males, 121 females; *M_age_* = 21.65, *SD_age_* = 2.40). Participants completed questionnaires in two time periods. At time 1, participants completed the measuring of humor production and demographic information. At time 2 (2 weeks later), participants completed the measuring of their creativity.

#### 2.1.2. Measure

**Humor production:** We measured participants’ humor production using the cartoon humor production paradigm (Brodzinsky & Rubien, 1976). First, the participants were told that cartoons usually consist of two elements, drawing and copywriting. Many cartoons are funny because the copywriting contains punchlines. Next, a cartoon was presented as an example, and then six pictures were shown to the participants. They were asked to look through each picture carefully in 2 min and write funny copy for each picture, making it into a humorous cartoon. Finally, we recruited two raters to score each answer of the participants on a 7-point scale (1 = *not humorous at all*, 7 = *very humorous*) according to the scoring criteria formulated by Ruch et al. (Ruch et al., 2009). For each cartoon, the humor score was the mean of two raters’ scores for the item.

**Creativity:** We used the daily creativity dimension of the *Kaufman Domains of Creativity Scale* to measure participants’ creativity (e.g., helping others cope with difficult situations), which consists of 11 items (Kaufman, 2012). Participants rated each item on a 7-point scale (1 = *strongly disagree*, 7 = *strongly agree*) (Cronbach’s α = 0.77).

**Control variables:** Age and gender were included as control variables in the study.

### 2.2. Results

Hierarchical regression was conducted to analyze the predictive effect of humor production on creativity. After controlling for age and gender, humor production was significantly positively associated with creativity: β = 0.349, *p* < 0.001 (see Table 1).

## 3. Study 2

Based on the results of Study 1, Study 2 aimed to explore the mediating role of self-efficacy in the relationship between humor production and creativity (Hypothesis 2).

### 3.1. Methods

#### 3.1.1. Participants

A power analysis (G* Power 3.1; Faul et al., 2009) showed that a sample size of 243 would be needed to achieve a power of 0.8 to detect an effect with a size of *f* = 0.04 and α = 0.05. We recruited 250 university students online via Credamo (https://www.credamo.com (accessed on 20 May 2022)); a professional online survey platform in China, which is similar to MTurk), and excluded 7 participants who failed the quality check, leaving a final sample of 243 participants (76 males and 167 females; *M_age_* = 21.59, *SD_age_* = 2.01).

#### 3.1.2. Measure

**Humor production:** Participants’ humor production was measured using the *Humor Efficacy Short Scale* developed by Silvia et al. and this scale mainly focuses on people’s beliefs on humor production (e.g., I think I can make almost anyone laugh), which consists of 12 items (Silvia et al., 2021). Participants rated each item on a 7-point scale (1 = *strongly disagree*, 7 = *strongly agree*) (Cronbach’s α = 0.87).

**Self-efficacy:** Participants’ self-efficacy was measured by *The General Self*-*Efficacy Scale* developed by Schwarzer and Jerusalem (e.g., it is easy for me to stick to my aims and accomplish my goals), which consists of 10 items (Schwarzer & Jerusalem, 1995). Participants rated each item on a 7-point scale (1 = *strongly disagree*, 7 = *strongly agree*) (Cronbach’s α = 0.91).

**Creativity:** The creativity was measured using the same scale as used in Study 1 (Cronbach’s α = 0.85).

**Control variables:** Age and gender were included as control variables in the study.

### 3.2. Results

#### 3.2.1. Descriptive and Correlative Analysis

The means, standard deviations, and correlations of the main variables are presented in Table 2.

Regression analysis was conducted using SPSS 26.0 to test the impact of humor production on creation. The results show that controlling for gender and age, humor production was positively related to creation (β = 0.271, *p* < 0.001), supporting Hypothesis 1. The pattern of results was identical without controlling for gender and age.

#### 3.2.2. Testing for the Mediating Effect

**Figure 1:**
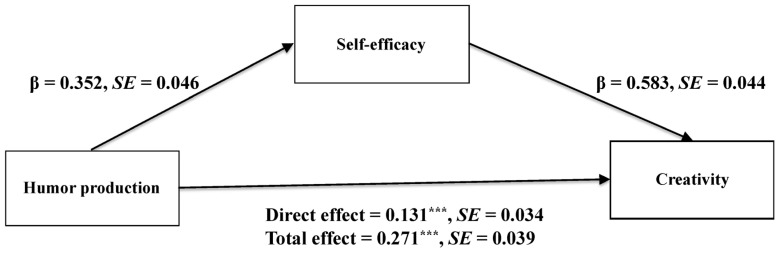
Regression results of the mediation model in Study 2 (humor production on creativity via self-efficacy; *** *p* < 0.001).

We used model 4 of PROCESS macro (Hayes, 2017) in SPSS to explore the mediating role of self-efficacy between humor production and creativity. The results are shown in Figure 1. Controlling for gender and age, humor production significantly positively predicted self-efficacy (β = 0.352, *p* < 0.001). After controlling for humor production, self-efficacy had a significant positive effect on creativity (β = 0.583, *p* < 0.001). And there was a significant indirect effect of humor production on creativity via self-efficacy (effect size = 0.140, 95%CI = [0.088, 0.196]), supporting Hypothesis 2. The pattern of results was identical without controlling for gender and age.

## 4. Study 3

Following previous studies (e.g., Ge & Hou, 2021; Jiang et al., 2020), Study 3 aimed to further explore the mediating role of self-efficacy in the relationship between humor production and creativity through a manipulation-of-mediation-as-a-moderator (MMM) design (i.e., manipulating self-efficacy). The design could provide both strong evidence for the causal relationship between self-efficacy and creativity, and direct evidence for the relationships between humor production and creativity (Ge, 2023). Moreover, following previous studies (e.g., Dai et al., 2025; Yin et al., 2025), Study 3 would change the measures of creativity to obtain robust results.

### 4.1. Methods

#### 4.1.1. Participants

A power analysis (G* Power 3.1; Faul et al., 2009) shows that a sample size of 225 would be needed to achieve a power of 0.8 to detect an effect with a size of *f* = 1.88 and α = 0.05. We recruited 265 participants online via Credamo and excluded 40 participants who failed the quality check questions, leaving a final sample of 225 participants (77 males and 146 females; *M_age_* = 28, *SD_age_* = 7.8).

#### 4.1.2. Procedure and Measures

After reading the informed consent, participants first answered the humor production scale (consisting of 12 items; e.g., I think I can make almost anyone laugh) as in Study 2 (Silvia et al., 2021) (Cronbach’s α = 0.70) and a personality questionnaire independent of study purposes. Then, participants were randomly assigned to either a high self-efficacy condition (*N* = 109) or a control condition (*N* = 116).

Participants in the high self-efficacy group were told, “*I am pleased to inform you! The personality questionnaire you just filled out is an indirect test of whether you have creativity, and we found that you have high creativity. In our sample survey, you can rank in the top 15%. Please describe your feelings in 30 words*.” In the control group, participants were not given feedback on their abilities, but were asked only: “*Please describe your experience the last time you did laundry in 30 words*.” This manipulation method has been used in previous studies (L. Hu et al., 2007; Jerome et al., 2002). Then, participants answered a question, “*Next, you will be asked to complete a task about creativity, do you have the confidence to complete it*” on a 7-point scale (1 = *not confident at all*, 7 = *very confident*) as the manipulation test.

Subsequently, all participants completed the self-efficacy questionnaire (Tierney & Farmer, 2002) and the Alternative Use Task (AUT) (Guilford, 1967). The AUT was used to measure creativity, and participants were asked to come up with as many creative uses for two AUT objects (cans and umbrellas, 2 minutes each) as possible in this study (Beaty & Johnson, 2021; Nusbaum et al., 2014). In Study 3, the objective scoring method proposed by Colzato et al. was used to score the novelty performance of participants in AUT tasks (Colzato et al., 2012). Taking cans as an example, firstly, the use of cans written by all the participants is summarized and organized to form an answer database. Secondly, the frequency of each answer is counted, and different scores are assigned to each answer according to the frequency. If an answer appears less than 1% in the answer database, two points are counted; if an answer appears less than 5% in the answer database, one point is counted. The sum of all the answers written by each participant is the participant’s novelty score on the topic. Finally, the score of the participant in the two questions is averaged, that is, the AUT score of the participant is obtained. There are two reasons why this method is suitable for this study. For one thing, this method is objective and avoids the subjective bias in the consensus assessment technique. For another, the number of participants in this study is more than 200, which meets the conditions to establish a certain scale of the answer database.

### 4.2. Results

#### 4.2.1. Manipulation Check

The manipulation check was successful. One-way ANOVA showed that the level of self-efficacy in the high self-efficacy group (*M* = 5.93, *SD* = 0.79) was significantly higher than that in the control group (*M* = 5.66, *SD* = 0.97), *F*(1, 223) = 4.938, *p* < 0.05.

#### 4.2.2. Testing for the Effect of Humor Production on Self-Efficacy

A stepwise regression analysis was conducted to test the effect of humor production on self-reported self-efficacy (confidence to complete the creative task). In Step 1, gender and age were included. In Step 2, humor production and the manipulated self-efficacy condition were included. In Step 3, the interaction term of humor production and the manipulated self-efficacy condition were included. The results reveal that humor production significantly positively predicted the level of self-efficacy measured after the priming phase. Self-efficacy manipulation did not moderate this effect, β = −0.07, *p* = 0.31. For the model of Step 3, *R*^2^ = 0.444, *F*(5, 219) = 37.00, *p* < 0.001. The results show that after the self-efficacy was manipulated, whether in the high self-efficacy group or the control group, the participants with a higher level of humor production were more likely to feel a higher level of self-efficacy.

#### 4.2.3. Testing for the Effect of Self-Efficacy on Creativity

After controlling for age, gender, and humor production, ANCOVAS was conducted to indicate that participants in the high self-efficacy condition (*M* = 2.99, *SD* = 1.86) had higher AUT scores than those in the control condition (*M* = 2.26, *SD* = 1.41), *F*(1, 223) = 10.94, *p* = 0.001, η^2^ = 0.05.

#### 4.2.4. Testing for the Moderating Effect

Moreover, as shown in Table 3, after age and gender were included as covariates, the regression model suggests that the interaction of humor production and manipulated self-efficacy positively predicted the creative scores of AUT, β = −0.187, *p* = 0.041.

**Figure 2:**
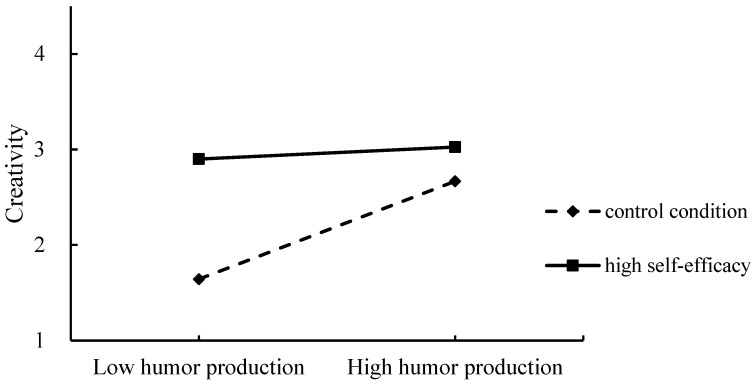
The moderating role of self-efficacy between humor production and AUT scores in Study 3.

We then conducted a simple slope test. The results in Figure 2 illustrate that, in the control condition, humor production positively predicted AUT scores, β = 0.68, *p* = 0.001, 95%CI = [0.28, 1.09], while in the high self-efficacy condition, such an effect vanished, β = 0.08, *p* = 0.69, 95%CI = [−0.33, 0.50], which further verified hypothesis 2 that self-efficacy partially explained the internal mechanism of the positive effect of humor production on creativity.

## 5. Study 4

Based on Studies 1–3, Study 4 aimed to explore the boundary condition of humor production affecting creativity, and further extend the theoretical model of this research to the workplace to enhance its ecological validity.

### 5.1. Participants

The study took place in three time periods. At time 1, 500 employees were recruited from Sojump (https://www.sojump.com (accessed on 30 June 2022)); another professional online survey platform in China like Credamo). Data on humor production and demographic information were collected. At time 2 (2 weeks later), 457 participants completed the follow-up questionnaires measuring their self-efficacy and fear of authority. At time 3 (a month later), 441 participants (51% male, *M_age_* = 29.16, *SD_age_* = 4.20) completed the questionnaires measuring their creativity.

### 5.2. Measures

**Humor production:** Humor production was measured by the humor production subscale of the *Multidimensional Sense of Humor Scale* (e.g., I’m regarded as something of a wit by my friends), which consists of 11 items (Thorson & Powell, 1993). Participants rated each item on a 7-point scale (1 = *strongly disagree*, 7 = *strongly agree*) (Cronbach’s α = 0.93).

**Self-efficacy:** The same general self-efficacy scale as in Study 2 was used (Cronbach’s α = 0.93).

**Fear of authority:** Fear of authority was measured by the *Scale of Fear of Authority* (e.g., I tend to defer to people with authority), which consists of 8 items (Zi & Zhou, 2006). Participants rated each item on a 7-point scale (1 = *strongly disagree*, 7 = *strongly agree*) (Cronbach’s α = 0.88).

**Creativity:** Creativity was measured by *The Runco Ideational Behavior Scale* (e.g., I think about ideas more often than most people), which consists of 23 items (Runco et al., 2001). Participants rated each item on a 7-point scale (1 = *strongly disagree*, 7 = *strongly agree*) (Cronbach’s α = 0.92).

**Control variables:** Age and gender were included as control variables in the study.

### 5.3. Results

#### 5.3.1. Descriptive and Correlative Analysis

The means, standard deviations, and correlations of the main variables are presented in Table 4.

#### 5.3.2. Testing for the Total Effect

Regression analysis was conducted using SPSS 26.0 to test the impact of humor production on creation. The results showed that controlling for gender and age, humor production was positively related to creation (β = 0.349, *p* < 0.001), supporting Hypothesis 1. The pattern of results was identical without controlling for gender and age.

#### 5.3.3. Testing for the Mediating Effect

A mediation effect test similar to that in Study 2 revealed that controlling for gender and age, humor production significantly positively predicted self-efficacy (β = 0.496, *p* < 0.001). After controlling for humor production, self-efficacy had a significant positive effect on creativity (β = 0.541, *p* < 0.001). There was a significant indirect effect of humor production on creativity (effect size = 0.285, 95% CI = [0.221, 0.354]), thus supporting Hypothesis 2.

#### 5.3.4. Testing for the Moderated Mediation Effect

The moderation model was tested by model 7 in PROCESS macro (Hayes, 2017). Gender and age were taken as control variables. It shows that the index of moderated mediation model of the effect of humor production on creativity was significant: Index of moderated mediation = −0.04, *SE* = 0.02, 95%CI = [−0.078, −0.001]. Among them, the interaction of humor production and fear of authority had a significant main effect on self-efficacy (β = −0.075, *p* = 0.047) (see Table 5).

**Figure 3:**
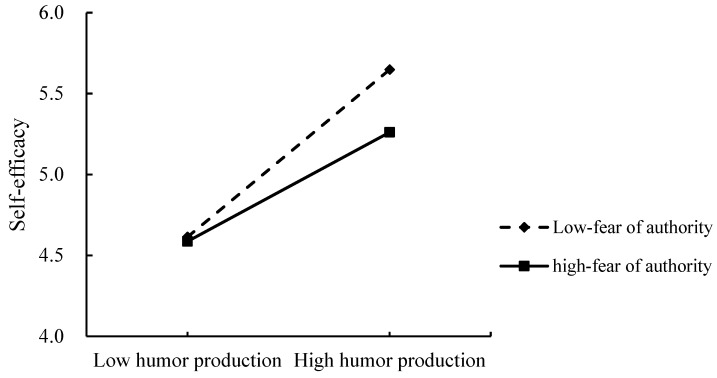
Fear of authority moderates the relationship between humor production and self-efficacy in Study 4.

The Simple Slope Test showed in the low level of fear of authority (−1 SD), humor production could positively predict self-efficacy (β = 0.57, *t* = 9.97, *p* < 0.001). However, in the low level of fear of authority (+1 SD), the relationship between humor production and self-efficacy was weakened (β = 0.37, *t* = 6.07, *p* < 0.001) (see Figure 3).

Moreover, we examined the conditional mediating role in different fear-of-authority levels (see Table 6). In a low level of fear of authority, the indirect effect of humor production on creativity through self-efficacy (β = 0.240, *SE* = 0.039, 95%CI = [0.166, 0.318]) is stronger than that in a high level of fear of authority (β = 0.156, *SE* = 0.034, 95%CI = [0.092, 0.226]), supporting Hypothesis 3.

## 6. General Discussion

The present research investigated the effect of humor production on creativity, as well as the mediating role of self-efficacy and the moderating role of fear of authority, through four empirical studies. Study 1, using two-wave data, revealed that humor production positively predicted creativity. Study 2, using a new sample, identified that self-efficacy mediated the relationship between humor production and creativity. Based on the findings of Study 1 and Study 2, Study 3 replicated the results by manipulating self-efficacy. Study 4, using a new sample and three-wave data, extended the conceptual framework to the workplace, and further found that fear of authority alleviated the indirect effect of humor production on creativity via self-efficacy.

### 6.1. Theoretical Implications

This research has several theoretical implications. First, we contribute to the literature on a sense of humor and creativity by exploring the impact of humor production, an active expression of humor, on human creativity. Although previous research has consistently identified a positive correlation between humor and creativity (Akben & Coskun, 2024; Jurčová, 1998; Kellner & Benedek, 2017), few studies have explored how the sense of humor affects creativity from the perspective of humor production (Ruch & Heintz, 2019). The present research extends the existing literature by empirically demonstrating that humor production exerts a positive influence on creativity. These findings align with the theoretical framework proposed by scholars (Galloway, 1994; O’Quin & Derks, 1997), establishing a substantial and direct connection between humor production and creativity.

Second, we contribute to the literature by understanding the mediating role of self-efficacy between humor production and creativity. Positive cognition (Černe et al., 2022) and positive emotion (Filipowicz, 2006; Isen et al., 1987) are beneficial for increasing one’s self-efficacy. The present research adopted different methods (self-report survey and experimental design), and found that self-efficacy is the internal mechanism of humor production to promote creativity. The results indicate that expressing humor not only brings positive effects on others but also returns to the humorous person through emotional contagion, allowing the humor maker to have a positive evaluation of themselves. When individuals believe that they can complete a task with positive emotion and cognition, their self-efficacy will be improved, which is the driving force to start and maintain creative activities (Amabile, 1983; Urban & Urban, 2025).

Third, we contribute to the literature by understanding the moderating role of fear of authority in the indirect effect between humor production and creativity, especially in the context of work. Previous research into the intersection of humor and creativity has predominantly concentrated on examining the association between leader humor and employee creativity (W. Hu & Luo, 2023). The present research mainly focuses on the effect of employees’ humor production on their creativity in corporate contexts. We found fear of authority alleviated the indirect effect of humor production on creativity, which is consistent with previous results and has enriched the relevant research on authoritarian leadership (Wang et al., 2013; Zhang et al., 2011). Moreover, such findings are also consistent with the characteristics of management style in a collectivist culture, where some employees tend to fear authority due to authoritarian leadership (Ahmad Bodla et al., 2019). The nurturing of creativity within an organizational context often necessitates employees’ ability to freely generate and contribute valuable ideas (Amabile, 1988). However, authoritarian leadership is associated with diminished employees’ external performance and the elicitation of negative emotions, such as fear and anger (Chen et al., 2014).

### 6.2. Practical Implications

Our research also has several practical implications. Creativity plays a vital role in enhancing customer satisfaction and loyalty, which is conducive to the survival and success of enterprises (Gumusluoglu & Ilsev, 2009; Shen et al., 2019). The findings from our research hold valuable implications for practical application, particularly in the context of selecting and developing creative talent within enterprises. For instance, the assessment of employees’ humor production abilities may serve as a predictive measure of their creative potential in future work endeavors.

Moreover, it is advisable to implement humor training initiatives, particularly within departments that demand a high degree of creativity from employees. For instance, the advertising creative team facing constant pressure for innovative campaigns could benefit from humor’s dual role in reducing cognitive rigidity and enhancing collaborative creativity. Additionally, it is important to acknowledge that employees’ fear of authority can hinder the expression of their humorous attributes, consequently impacting their creative potential. Given that, we suggest that leaders could actively create an atmosphere of equal interaction with employees and even use a humorous style to communicate with them. This approach can effectively mitigate employees’ fear of authority, thereby unleashing their capacity for humor production and subsequently enhancing their overall creativity. For example, leaders could cultivate their transformational leadership style (i.e., being friendly and approachable to others). Transformational leadership could promote employees’ internal motivation and work passion and reduce their pressure (Bass, 1995), which might decrease employees’ fear of authority.

### 6.3. Limitations and Future Directions

This study has some limitations that should be considered. First, it is noteworthy that humor production is not confined to purely social contexts but extends to non-social settings as well. In social environments, humor production encompasses activities aimed at amusing others, involving the use of jokes or other humorous expressions. In contrast, within non-social environments, humor production entails actions undertaken for personal amusement without any direct intention to entertain others (Ruch & Heintz, 2019). Our study focused on the impact of humor production on creativity within the social context, leaving the potential influence of humor production in non-social environments. We recommend that future research could provide a more comprehensive understanding of the dynamics involved.

Second, our sample was limited to Chinese participants, although we utilized multiple data sources to improve the sample representativity (e.g., using two-wave data, experimental design). However, authoritarian leadership is more prevalent in Chinese organizations, but not in Western organizations (Pellegrini & Scandura, 2008). In this line, future research is expected to further explore humor production and creativity across multiple cultures to provide a better understanding of the potential boundary conditions of cultural differences (Cao et al., 2023).

Third, while some studies have established the positive effect of humor appreciation on creativity (Huang et al., 2015; Lang & Lee, 2010; Ziv, 1983), it is important to acknowledge that the present research primarily concentrated on humor production and did not incorporate measurements of participants’ humor appreciation. Future research could compare the effect sizes between humor appreciation, humor production, and creativity.

Fourth, we explored why and when humor production impacts creativity. On the one hand, we only identified the mediating role of general self-efficacy rather than creative self-efficacy. Future studies are recommended to identify the mediating role of creative self-efficacy between humor production and creativity. On the other hand, we only verify the moderating role of fear of authority in the relationship between humor production and self-efficacy. However, fear of authority might influence people’s humor production. For example, employees with a high fear of authority might be afraid of producing humor in the workplace, avoiding leaving a negative impression on superiors. Future studies are recommended to explore the relationship between fear of authority and humor production.

## 7. Conclusions

Our study investigated how, why, and when humor production influences human creativity. The findings could be included as follows. Firstly, humor production is positively associated with creativity. Secondly, humor production could enhance individuals’ self-efficacy, which in turn increases their creativity. Thirdly, the fear of authority moderates the mediating role of self-efficacy in the relationship between humor production and creativity. Specifically, compared to those with a high fear of authority, humor production is more likely to increase individuals’ creativity via self-efficacy with a low fear of authority.

## Figures and Tables

**Table 1:** Results of multiple regression analysis in Study 1.

	Creativity	Creativity
	β (*SE*)	β (*SE*)
Gender	0.028 (0.103)	0.019 (0.097)
Age	−0.196 ** (0.020)	−0.203 ** (0.018)
Humor production		0.349 *** (0.165)
*R* ^2^	0.028	0.146
*F*	3.494 *	10.893 (<0.001)

Note: *N* = 175. * *p* < 0.05; ** *p* < 0.01; *** *p* < 0.001.

**Table 2:** Descriptive statistics and correlations of the study variables in Study 2.

	*M*	*SD*	1	2	3	4
1. Gender	—	—	1			
2. Age	21.59	2.01	−0.050	1		
3. Humor production	4.687	1.090	−0.121	−0.053	1	
4. Self-efficacy	4.852	0.852	−0.208 ***	−0.074	0.376 ***	1
5. Creativity	5.042	0.746	−0.245 ***	−0.055	0.423 ***	0.676 ***

Note: *N* = 243. *** *p* < 0.001.

**Table 3:** Results of multiple regression analysis in Study 3.

	Creativity (AUT Scores)		
	β (*SE*)	β (*SE*)	β (*SE*)
Gender	−0.093 (0.236)	−0.036 (0.235)	−0.036 (0.234)
Age	−0.070 (0.014)	−0.049 (0.014)	−0.045 (0.014)
Humor production		0.171 * (0.150)	0.302 ** (0.207)
Self-efficacy group		0.218 ** (0.222)	−0.219 ** (0.220)
Humor production × Self-efficacy			−0.187 * (0.291)
*R* ^2^	0.004	0.064	0.077
*F*	1.441	4.819 **	4.761 ***

Note: *N =* 225. Self-efficacy group: 0 = control group, 1 = high self-efficacy group. * *p* < 0.05; ** *p* < 0.01; *** *p* < 0.001.

**Table 4:** Descriptive statistics and correlations of the study variables in Study 4.

	*M*	*SD*	1	2	3	4	5
1. Gender	—	—	1				
2. Age	29.16	4.20	0.057	1			
3. Humor production	5.313	0.692	0.003	−0.040	1		
4. Self-efficacy	5.043	0.942	0.008	−0.006	0.496 ***	1	
5. Fear of authority	4.233	1.081	0.182 ***	0.072	−0.181 ***	−0.200 ***	1
6. Creativity	4.573	0.732	0.021	−0.013	0.349 ***	0.607 ***	0.102 *

Note: *N* = 433. Gender: 0 = male, 1 = female; * *p* < 0.05; *** *p* < 0.001.

**Table 5:** Results of multiple regression analysis in Study 4.

	Self-Efficacy		Creativity	
	β	*SE*	β	*SE*
Gender	0.026	0.080	0.018	0.056
Age	0.019	0.009	−0.005	0.007
Humor production	0.465 ***	0.058	0.136 **	0.035
Fear of authority	−0.112 *	0.038		
Humor production × Fear of authority	−0.075 *	0.037		
Self-efficacy			0.541 ***	0.034
*R* ^2^	0.255		0.383	
*F*	30.786 ***		66.322 ***	

Note: *N =* 433. * *p* < 0.05; ** *p* < 0.01; *** *p* < 0.001.

**Table 6:** Conditional effect of fear of authority on the mediation in Study 4.

Fear of Authority	β	*Boot SE*	*Boot LLCI*	*Boot ULCI*
Low fear of authority (−1 SD)	0.240	0.039	0.166	0.318
Mean fear of authority	0.198	0.030	0.141	0.259
High fear of authority (+1 SD)	0.156	0.034	0.092	0.226

## Data Availability

The data presented in this study are available on request from the corresponding author (the data are not publicly available due to privacy or ethical restrictions).
